# Treatment of a Localized Stage III Periodontitis in the Esthetic Zone with Guided Tissue Regeneration Technique on a Heavy Smoker Patient with 12-Year Follow- up: A Case Report

**DOI:** 10.30476/dentjods.2024.100483.2224

**Published:** 2024-06-01

**Authors:** Fatme Mouchref Hamasni, Fady El Hajj

**Affiliations:** 1 Former Head of Dept. of Periodontology and Director of the Master Program, Lebanese University Faculty of Dental Medicine Beirut Lebanon, Beirut Arab University, Faculty of Dental Medicine, Beirut, Lebanon; 2 Former Head of Dept. of Periodontology, Lebanese University Faculty of Dental Medicine. Beirut, Lebanon, Lebanese University, Faculty of Dental Medicine, Beirut, Lebanon

**Keywords:** Guided tissue regeneration, Esthetics, Smoking

## Abstract

This case report exhibits a heavy smoker female patient with a localized stage III periodontitis who has been under the smoking cessation program during the pre-surgical period, followed by a strict maintenance program for the past twelve years, after being treated with guided tissue regeneration techniques and restored with zirconia prosthetic crowns. A 50-year-old, heavy smoker (> 40 cigarettes per day), systemically healthy female patient presented complaining of mobility and pain in the upper right central incisor, which was temporarily splinted to the left central incisor using resin composite. After clinical and radiographic examination, significant damage of the attachment apparatus, deep periodontal lesions extending the middle portion of the root, and severe infrabony defect were noted. Following the initial hygienic phase, a guided tissue regeneration surgery using xenograft bone substitute covered by a resorbable collagen membrane was performed. After six months of healing, four zirconia crowns were cemented on the central and lateral incisors based on patient esthetic compliance. During the 12-year follow-up period, neither residual pockets nor gingival recession were observed, and perfect marginal bone stability, and esthetic and functional results were noted. This case shows the predictability of a conservative surgical technique, the guided tissue regeneration, based on appropriate treatment planning and a strict maintenance program. It also demonstrates the importance of at least a 6-month healing period after such surgeries, allowing complete tissue maturation and a re-establishment of the supra osseous gingival tissues in order to locate the prosthetic margins without interfering with the soft tissues integrity

## Introduction

Periodontitis is defined as a chronic multifactorial inflammatory disease associated with dysbiotic plaque biofilms, and characterized by progressive destruction of the tooth‐supporting apparatus manifested through clinical attachment loss, alveolar bone loss, and presence of periodontal pocketing [ 1 ]. 

Knowing that the treatment of periodontitis is based on patient-related factors, the treatment of such periodontal disease generally consists of two phases including (1) Preventing disease progression through active therapy, and (2) Supporting patients in maintaining a healthy periodontium [ 2 ].

The management of periodontal clinical cases requires, primordially, a non-surgical phase that consists of oral hygiene instructions with control of local irritating factors and systemic risk factors [ 3 ], followed by the elimination of biofilms and mineralized deposits [ 4 ], supported when needed by systemically antimicrobial drugs [ 5 ]. After evaluation of the soft tissue’s response and behavior, surgical approaches may be indicated to eliminate residual pockets and/or create a gingival morphology that allows the patient to self-elimination of the plaque [ 2 ]. The technique of guided tissue regeneration using resorbable or non-resorbable membranes may be applied in order to regenerate periodontal defect, depending on the morphology of the defect and patient cooperation. 

The aim of such procedures is to rebuild the tooth-supporting structures, including the cementum, periodontal ligament, and alveolar bone, using a cell-dense membrane that can isolate the defects, providing a space for the blood clot to permit the slow-proliferation of periodontal components [ 6 ].

Accomplishing regenerative therapy is well documented in the literature and depends on numerous aspects such as scientific-based choice of bone substitutes and barrier membranes, suture techniques and removal time, and adequate post-surgical control. However, the key of success of this technique is the early and safe wound closure, especially in the first post-surgical weeks [ 7 - 8 ].

This minimizes the risk of membrane complications by avoiding smoking, providing an undisturbed healing period that allows the complete re-establishment of the supraosseous soft tissue complex represented by the epithelial and connective attachment and the gingival sulcus, and finally, strict maintenance therapy [ 9 ]. 

## Case Presentation

### Patient History and Chief Complaint

A 50-year-old heavy smoker (more than 40 cigarettes per day) and healthy female patient, an instructor at the Lebanese University, living a hundred kilometers from our office, was referred to our clinic in 2009 for the evaluation of her two upper central incisors. The patient’s dentist had recommended the extraction and immediate implantation of these two compromised teeth after the failure of the restorative treatment and an acquired open central diastema. The patient’s chief complaint was that she did not like her smile, she was afraid to bite on her anterior teeth, and she was not satisfied with the esthetic results previously performed. 

### Initial Assessment

**Figure 1:**
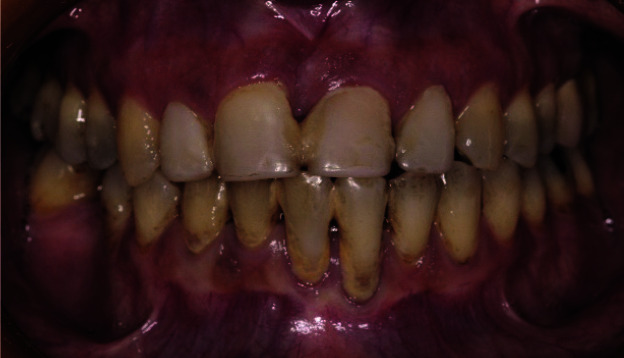
Frontal view showing plaque and calculus accumulations, gingival inflammation, and periodontal complications around all teeth

**Figure 2:**
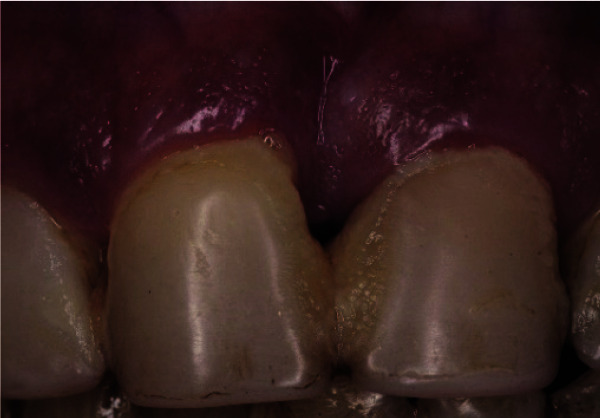
Clinical photo of both central incisors with an acquired diastema and anesthetic restorative treatment

**Figure 3:**
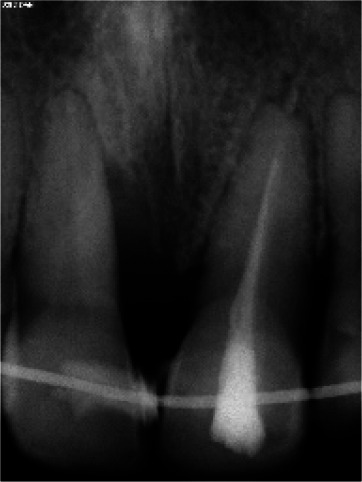
A vertical bone destruction extending to the third part of the root at the mesial site of the right incisor is visible on a peri-apical radiograph

**Figure 4:**
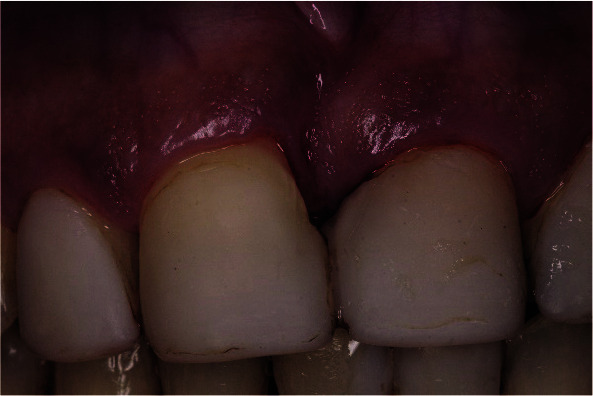
Clinical photo assessed the periodontal soft tissues status 3 weeks after scaling and root planning

Diagnostic and thorough clinical examination, which included plaque index, bleeding index, and tissue biotype evaluation revealed insufficient oral hygiene with plaque and calculus accumulation and the presence of periodontal complications all over the teeth (Figures 1-2). At the level of the upper anterior maxilla, a slight inflammation of thick periodontal tissues was noticed. The measurements of the sulcus depth and attachment level of both central incisors at the mesio-buccal, mid-buccal, disto-buccal, disto-palatal, mid-palatal, and mesio-palatal sites exhibited the presence of a localized periodontal pocket with more than 10 mm of probing depth at the mesial site of the right central incisor (tooth number 11). The concerning tooth presented a slight bucco-palatal mobility limited to 2 mm. Radiographic examination (Figure 3) showed advanced vertical bone resorption at the mesial side of tooth number 11, extending to the third apical part of the root. A mechanical supra gingival scaling and subgingival root planning were performed and a mouthwash (0.1% chlorhexidine and 0.5% chlorobutanol) was prescribed for a period of 10 days to assess the periodontal tissue response. The patient expressed that it was impossible for her to stop smoking, as she has tried several times without result, and she has resumed smoking each time. After several education sessions on the harmful effect of smoking on tissue healing after surgery, she accepted to follow the smoking cessation program, which consisted of stopping smoking one week before surgery and three weeks post-operatively. The patient returned for surgical treatment after 3 weeks (Figure 4).

### Surgical Treatment and follow-up

Following local anesthesia, a submarginal scalloped incision on the buccal and palatal aspects of the four upper incisors was made, extended to one tooth mesially and distally, allowing the elimination of the sulcular epithelium. Care was taken to confine the submarginal incision within the buccal gingival tissue so that the buccal keratinized gingiva was preserved within the flap. Special attention was paid to preserve the height and the mesio-distal width of the interproximal papilla between the two central incisors in order to facilitate maximal coverage of the treated zone during wound closure.

**Figure 5:**
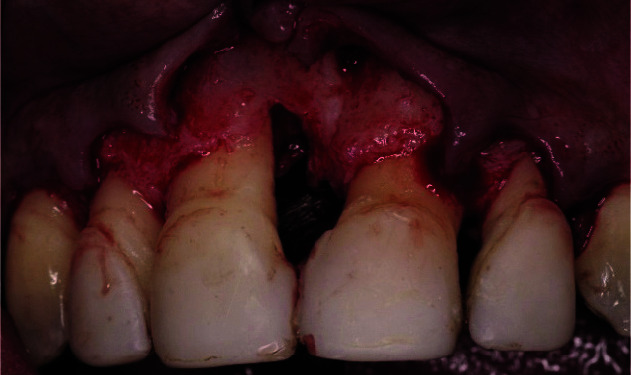
Vertical bone resorption at the mesial aspect of the right incisor was identified when performing buccal and palatal flap elevation

**Figure 6:**
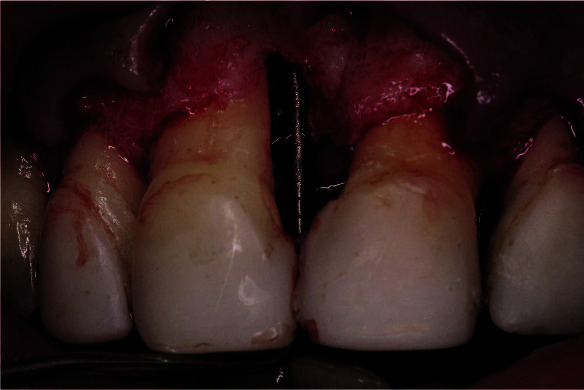
Measurement of the bone defect showing a 2mm depth of a 3-wall defect

**Figure 7:**
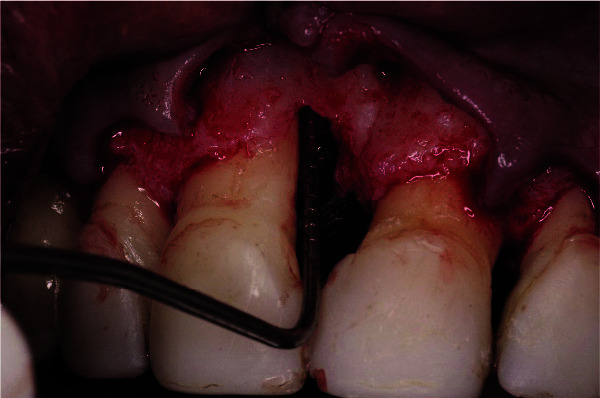
Measurement of the bone defect showing a 3 mm depth of a 2-wall defect

**Figure 8:**
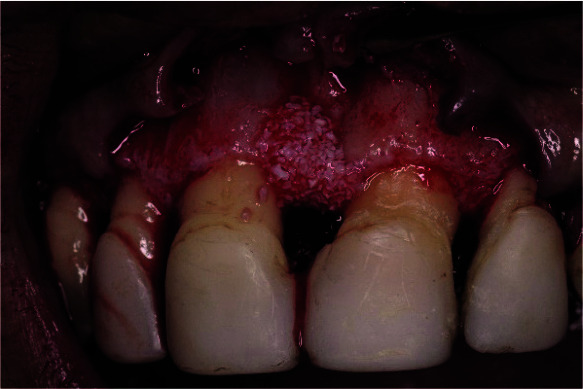
A bovine bone substitute (Bio-Oss) filling the bone defect at the mesial site of tooth number 11

**Figure 9:**
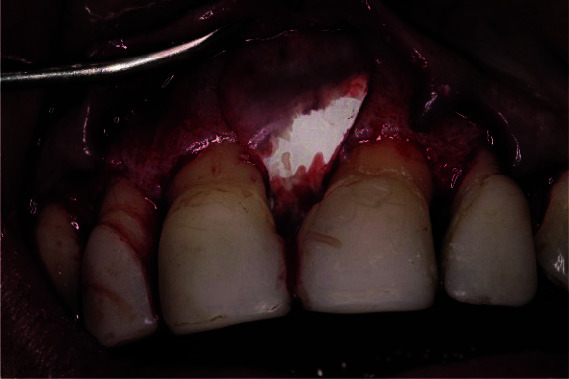
A Collagen membrane (Bio- Gide) adapted in place, covering the defect

**Figure 10:**
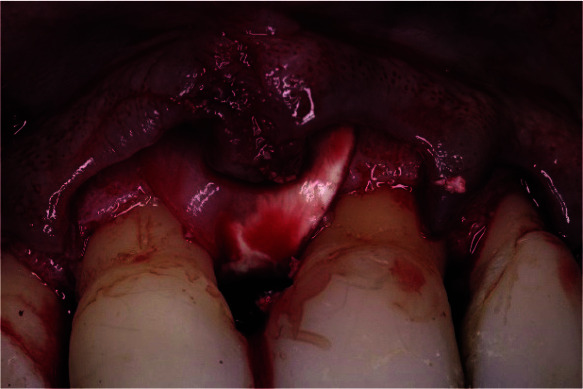
Flap adaptation, note the presence of an intact papilla between the incisors allowing a complete coverage of the membrane

**Figure 11:**
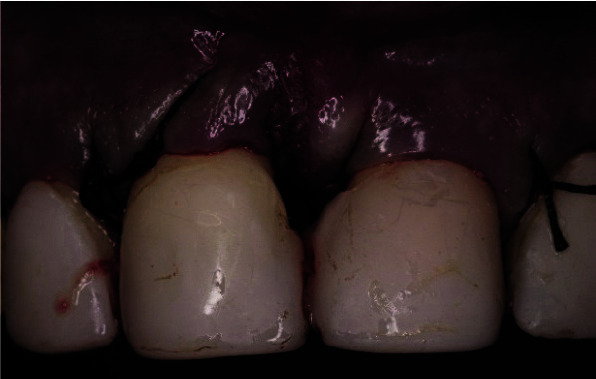
Photo showing a tension free flaps adaptation with interrupted single sutures

The buccal flap was elevated full thickness to expose 2 to 3mm of alveolar buccal bone, followed by a split thickness in order to facilitate a coronal displacement of the flap itself. The palatal flap was elevated full thickness. Elevation of flaps exposed a deep vertical bone resorption at the mesial aspect of the right incisor (Figure 5), with a 2mm depth of a three-wall defect in the apical part (Figure 6), a 3 mm depth of a two-wall defect in the middle side (Figure 7), and a 2mm depth of a one-wall defect in the coronal part. Root surfaces were slightly scaled using ultrasonic and hand curettes, then the technique of guided tissue regeneration was applied using xenograft bone substitute Bio-Oss^®^ (Figure 8), covered by a resorbable bilayer collagen membrane (Geistlich biomaterial Bio-Gide®) trimmed and adapted without the need for fixation tools (Figure 9). The buccal and lingual flaps were subsequently positioned coronally, ensuring maximum coverage of the membrane (Figure 10), using interrupted single sutures with 5-0 monofilament non-absorbable polyamide (Ethicon^*^ US) (Figure 11). An association of amoxicillin 500 mg and metronidazole 250 mg was prescribed three times a day for eight days with analgesics for the first two days or as needed. The patient was instructed to rinse with a 0.2% chlorhexidine solution three times a day for 15 days, starting 24 hours post-operatively.

**Figure 12:**
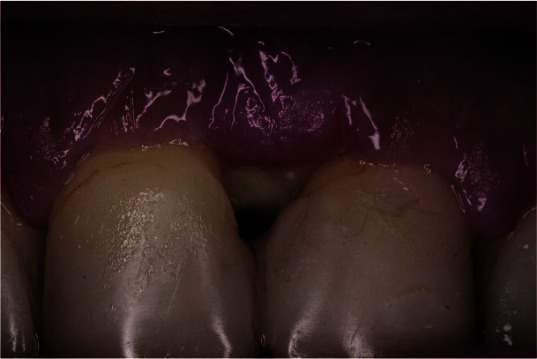
Clinical view 20 days post-surgery showing 2mm of membrane exposure

**Figure 13:**
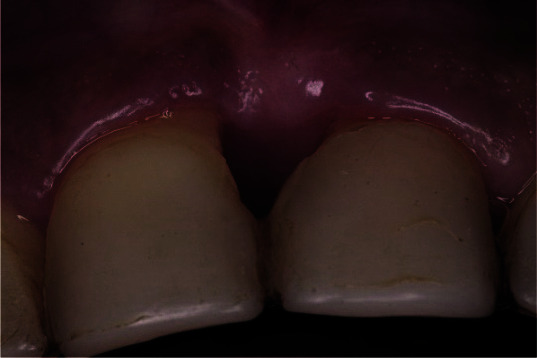
Three months post-operatively, complete soft tissue healing with perfect continuity of the papilla between two central incisors is noted

**Figure 14:**
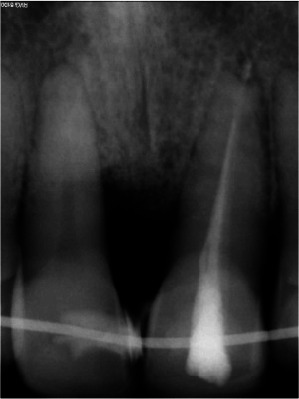
A control radiograph at 3 months post-surgery evaluating the volume stability of the added bone

Sutures were removed 10 days following the surgery, and the patient was scheduled for a recall visit every two weeks for prophylaxis. Ten days after sutures removal and before the first assessment appointment, the patient mentioned that something white was coming out of the gingiva and urgently asked for an examination. At that visit, 20 days post-surgery, the clinical exam revealed a 2 mm of membrane exposure without any sign of infection (Figure 12). A reinforcement of oral hygiene with a local ointment with chlorhexidine 0.2% was suggested, and the follow-up appointment was cut down to once a week. Three months post-surgery, clinical and radiographical assessments showed excellent soft tissue healing (Figure 13) and perfect hard tissue stability (Figure 14). The patient was asked to keep following the oral hygiene instructions and to return after 3 months for prosthetic rehabilitation. No modification of tooth preparation or of prosthetic margins of the temporary restoration was made during the 6-month post-operative period, leading to the complete maturity of the supra osseous soft tissues and the stability of the gingival margin. At 6 months, a four-unit zirconia bridge was performed based on patient esthetic compliance, the case was documented clinically and radiographically, and a follow-up maintenance program was scheduled.

### Maintenance and Supportive therapy

As the patient returns to smoking one pack a day, a strict maintenance program was imposed during the last 12 years. A supportive periodontal treatment based on professional plaque elimination was performed every 3 to 4 months for the first two years after surgery, then twice a year as a regular control visit. 

**Figure 15:**
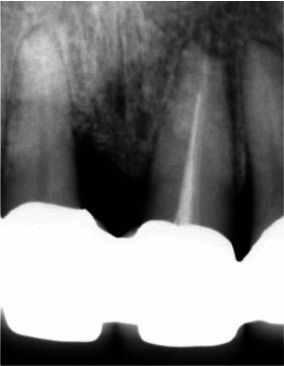
Peri-apical radiograph at 2 years post-surgery, with a substantial stability of the grafted bone, and significant adaptation of the prosthetic elements

**Figure 16:**
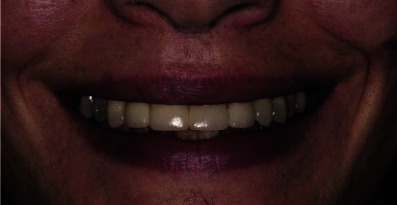
Clinical photos at 2 years post-operatively showing the respect of an esthetic smile

**Figure 17:**
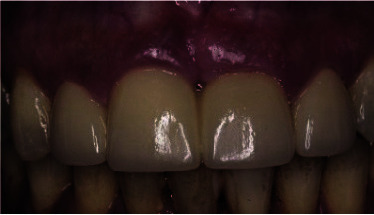
Clinical photo at 2 years post-operatively showing the respect of a soft tissues contour with adequate prosthetic crown integrations

**Figure 18:**
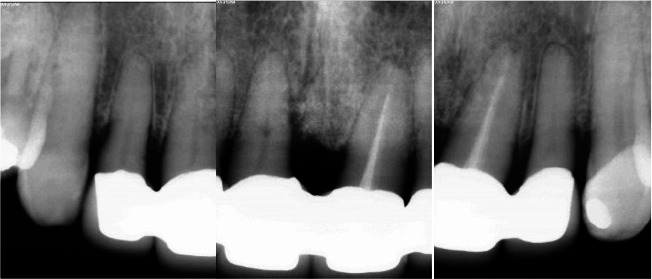
Radiographic evaluation 12 years post-surgery, no change in bone aspect or volume is noticed

**Figure 19:**
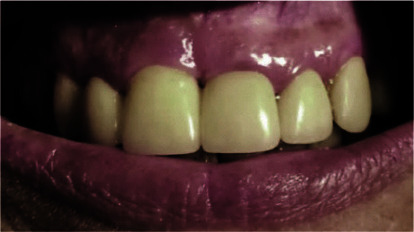
A healthy soft tissue with respect of the marginal contour is still maintained after 12 years of guided tissue regeneration technique and prosthetic performance

The first periapical follow-up radiograph was taken at 2 years post-surgery, showing the volume stability of the added bone (Figure 15). Clinical photos showed a good esthetic outcome (Figure 16) as well as a perfect soft tissue contour (Figure 17). Since the treatment was accomplished, neither the inflammatory conditions, nor the residual pockets were observed and no use of antibacterial or antiseptic mouth rinses or systemic antibiotics was needed. At 12 years post- surgery, radiographical and clinical examinations showed marginal bone stability (Figure 18) and good soft tissue integrity of the treated area (Figure 19), with perfect adaptation of the prosthetic crowns. 

## Discussion

The use of the regenerative technique and the conservation of a compromised tooth in the esthetic zone were highly appreciated by the patient. Aside from the esthetic and functional satisfactory results, this case showed a perfect clinical outcome 12 years after performing guided tissue regeneration techniques on a 7mm localized infrabony pocket. Besides, this procedure was performed on a heavy smoker patient, presenting a localized stage III periodontitis defined as significant damage to the attachment apparatus with deep periodontal lesions that was extended to the middle portion of the root associated with infrabony pockets [ 1 ]. Knowing the limited success of dental implants, in smoker patients [ 10 ] and/or patients presenting periodontal diseases [ 11 ], and the sensitive technique of replacing a single tooth in the esthetic zone [ 12 ], the minimally invasive regenerative technique remains a technique of choice when a right diagnostic, perfect surgical performance and strict follow-up program are established. These findings are in concordance with the recently published results supporting the long-term success of guided tissue regeneration therapy performed on deep intra-bony defects with 26-year follow-up [ 13 ]. The success and stability of such results may be related to two following main factors. The first factor is the smoking cessation program in order to reduce the risk of wound-related complications and to prevent severe ill effects such as ischemia and hypoxia of the tissues through the vasoconstrictor effect of nicotine [ 14 ]. The second factor is the undisturbed time of healing for the corono-apical height of the supraosseous gingival tissues, evaluated at 6 months post-surgery as a minimum period for the re-establishment of its dimension post-surgically, especially at the interproximal sites [ 15 ].

Such a research findings was mentioned in 2019 regarding the effects of patient- and surgery-related factors on supracrestal tissue reestablishment after crown lengthening procedure [ 15 ]. Results showed that the mean apicocoronal dimension of supracrestal gingival tissue was re-established 6 months postsurgically at all sites in the experimental and adjacent teeth, indicating that when performing crown lengthening procedure, the genetically preset supraosseous gingival tissues height should be considered as a guideline during per-surgical and post-surgical phases [ 15 ].

This latter was respected in the present case, which may be the main positive factor of this long-term stability observed, as no prosthetic procedure or any tooth modifications were done during the 6-month post-operative period leading to the complete re-establishment and stability of the supraosseous gingival tissues on the desired sites. Therefore, the prosthetic margins of the future crowns can be ideally located without violating the biological width or being at risk of complications leading to anesthetic outcomes.

The patient demonstrated full satisfaction and cooperation and gave her consent to the publication of the case.

## Conclusion

In periodontology, beside the perfect ability and knowledge of the principles of surgery, the key to the success of surgical therapy is a customized-patient-surgical technique guided by patient’s general status and habits, oral hygiene, tissue biotype, and specially the respect of an undisturbed post-operative period for tissue healing, allowing the establishment of an ideal esthetic outcome around the prosthetic component. 
